# Seroprevalence of non-typhoidal *Salmonella* disease and associated factors in children in Mukuru settlement in Nairobi County, Kenya

**DOI:** 10.1371/journal.pone.0288015

**Published:** 2023-07-17

**Authors:** Schola K. Peter, Joshua M. Mutiso, Mercy Ngetich, Cecilia Mbae, Samuel Kariuki

**Affiliations:** 1 Department of Zoological Sciences, Kenyatta University, Nairobi, Kenya; 2 Centre for Microbiology Research, Kenya Medical Research Institute, Nairobi, Kenya; 3 Wellcome Trust Sanger Institute, Cambridge, United Kingdom; Public Health England, UNITED KINGDOM

## Abstract

Non-typhoidal *Salmonella* (NTS) infections remain a significant public health challenge especially in sub-Saharan Africa. NTS disease is endemic in Kenya and is associated with sporadic fatal outbreaks in several regions of the country with poor resource setting. Data is limited on background exposure of NTS in the population in endemic areas and the general immune status of the community most affected by NTS. The aim of the study was to determine the proportion of children exposed to *Salmonella* Enteritidis or *Salmonella* Typhimurium O antigen among the apparently healthy children and patients and the associated host and environmental factors among children attending selected healthcare facilities in Mukuru, Nairobi County, Kenya. A cross-sectional case-control study was conducted among patients and apparently healthy participants aged 0–5 years. Blood was collected and centrifuged to obtain serum. The serum was used to test for the presence of antibodies (IgA, IgG, IgM) against NTS using ELISA. A questionnaire was administered to obtain relevant demographic, socio-economic and healthcare utilization information. A total of 382 children were recruited into the study. The NTS seroprevalence was 12.6%. Among the apparently healthy participants, mean age of those exposed to NTS was 36 months and those not exposed was 27 months. Among patients, the mean age was 39 months and those not exposed was 30 months. The seroprevalence of NTS infection among the apparently healthy was significantly associated with cooking water, washing water and age of the child. Treating water using chlorine or boiling method was identified as being protective against contracting *Salmonella* Typhimurium/Enteritidis. Among the patients, the proportion of exposure was significantly associated with keeping animals and the chicken count. There is a high exposure to NTS among young children below five years of age and the population has developed immunity to the disease.

## Introduction

Non-typhoidal *Salmonella* infections remain a significant public health problem worldwide in both industrialized and developing countries. *Salmonella enterica* that cause human disease is divided into non-typhoidal *Salmonella* (NTS) and typhoidal serotypes causing typhoid fever [1]. Since gastroenteritis typically caused by nontyphoidal *Salmonella* serovars are self-limiting, there is emergence of invasive nontyphoidal strains (iNTS) that cause severe systemic infection [2, 3]. The most common cause of nontyphoidal *Salmonella* infections are *Salmonella* enterica serotype Enteritidis and *S*. Typhimurium [4]. In most western countries, *S*. Enteritidis and *S*. Typhimurium have emerged as predominant serotypes in the last few decades [5]. Non-typhoidal *Salmonella* (NTS) commonly causing bacterial enteritis in humans, causes 93.8 million cases of gastroenteritis globally each year, with 155,000 deaths [6, 7]. Low-income countries are said to be at greater risk of infection with NTS due to poor hygiene and lack of access to safe water and food [8] while people living in developed countries are at greater risk due to the consumption of contaminated meat, fresh vegetables and fruits [9].

In most cases, nontyphoidal *Salmonella* infection is limited gastroenteritis that is uncomplicated and only requiring antimicrobial treatment [10]. In other cases, non-typhoidal *Salmonella* cause invasive infections such as blood stream, focal infections and sometimes meningitis by invading sterile sites [11]. Immunocompromised individuals such as HIV and malaria infected persons and malnourished children are at the greatest risk of getting infected with the iNTS disease [12]. It is estimated that 65% of iNTS cases in SSA occur in children <5 years of age, with 568/100,000 cases of person-years of observation (pyo) [13]. NTS disease is characterized by abdominal pain, diarrhea, nausea, vomiting and fever. Presentation of the non-specific febrile illness makes presumptive diagnosis and treatment a challenge for the clinician in low resource setting [6, 14]. Increase in *Salmonella* abundance has been reported to be associated with climatic conditions such as increased rainfall and temperature [15–17].

*Salmonella* bacteria is spread by the fecal-oral route, especially through the eating or drinking contaminated food and water, also by direct animal contact and sometimes from person to person [18]. Incubation period of invasive non-typhoidal *Salmonella* is typically 6–72 hours with an average of 12–36 hours. The illness lasts 4–7 days and most NTS patients recover from the disease without treatment [19].

Non-typhoidal *Salmonella* infection leads to intense antibody response against both protein antigens and non-protein antigens such as lipopolysaccharides (LPs). During the different stages of *Salmonella* infection, the antibodies perform different functions of protection. Immunoglobulin (Ig) M and IgA blocks penetration of *Salmonella* into deeper tissues [3]. Antibodies normally enhance bacterial engulfment via Fc receptor-mediated phagocytosis after bacterial migration from the intestine into the Peyer’s patches, mesenteric lymph nodes, spleen and liver, and this is due to the existence of Fc-receptor -mediated uptake that activates macrophages, thereby increasing their bactericidal activities. Fc-receptor and complement receptor-mediated uptake enhance phagocytosis of *Salmonella* and clearing of bacteria from serum through opsonization [3, 20]. Immunoglobulin A is a predominant antibody type in mucosal secretions, human colostrum, and breast milk and plays a key role in protecting the intestinal epithelium of newborns from invasive enteric pathogens like the Gram-negative bacterium such as non-typhoidal *Salmonella* [3, 21].

Apparently healthy individuals who could be carriers of NTS, are thought to be a major reservoir and source for dissemination of the NTS infection [22]. Mukuru informal settlement where this study was conducted is characterized by poor sanitation, dense population and unreliable water supply. These are elements that create an environment favorable for rapid dissemination of enteric infections and other sanitation-related pathogens through contaminated food and water [23]. This cross-sectional study reports the seroprevalence and the host and environmental factors found to be associated with NTS infection among children aged 0 to 5years in Mukuru informal settlement in Nairobi County.

## Materials and methods

### Study area

Mukuru informal settlement, located about 15 KM East of Nairobi city center is one of the largest slums in Nairobi County. It has a population of around 250,000 people [24]. High incidence of infectious diseases and increased mortality among those under five years is contributed by insufficient and unclean water sources, overcrowding, inadequate sanitation, poor drainage and solid waste disposal due to the use of public pit latrine [23]. Majority of the Mukuru slum dwellers are casual laborers working in the manufacturing industries situated near the slums, others are hawkers of different items while others run small scale business. Mukuru Informal settlement is divided into eight villages; Mukuru Sinai, Mukuru Lunga-Lunga, Kosovo, Mukuru kwa Reuben, Mukuru Kayaba, Mukuru North and Mukuru kwa Njenga. The study was conducted in hospitals in Mukuru kwa Njenga and Mukuru kwa Ruben villages. The three selected hospitals were Mukuru Ruben Centre (MR), Mukuru city council (MCC) clinic and Medical missionary of Mary (MMM) clinic.

### Study design, sample size and study subjects

The study adopted cross-sectional case-control study design. The sample size was calculated as per Sakpal (2010) [25] formula N = [(Zα/2 + Zβ^)2^ × {(p1 (1-p1) + (p2 (1-p2))}]/ (p1—p2)^2^. The prevalence of NTS in children in Mukuru slums of Kenya was estimated at 4% [23]. There being no any other seroprevalence study that had been conducted in the region, the proportion of children exposed to NTS among the symptomatic children was assumed to be 50% and a clinically important exposure difference of 10% as compared to the asymptomatic children was acceptable. A power of 80% was assumed at 5% significance level for a two sided test. N was the total sample size required for both populations. Considering 0.5 proportion of patients (P1), 0.4 proportion of NTS apparently healthy children (P2), desired level of statistical significance (Zα/2) of 1.96, desired power (Zβ) typically 0.84 for 80% power. P1-P2 = was the desired effect size (or difference of clinical importance). 191 consecutive patients presenting for care at three community clinics were approached for participation in the study if they were aged five years or below on the presentation day; had symptoms of diarrhea, fever or vomiting; had axillary temperature of at least 37.5°C; was a resident in the catchment area and their parents or guardians provided an informed consent form. 191 apparently healthy children attending the mother and child healthcare clinics (MCH) for routine vaccinations were consecutively recruited into the study if they were residents in the catchment area; were aged five years or below, did not report history of fever for the last three days and their temperature was below 37.5°C on the presentation day; did not show diarrhea or vomiting symptoms and their parents or guardians gave signed informed consent form. The participants were recruited into the study regardless of their gender.

### Questionnaire administration and specimen collection

A questionnaire was administered to the parents and guardians of the eligible study participants. The questionnaire helped to collect data on host and environmental factors associated with NTS. Relevant demographic, socioeconomic factors such as how many family members are there in the household, animals kept in the household, water, sanitation and hygiene (WASH) factors and healthcare utilization information was collected using structured questionnaires (S1 File). The questionnaire had a section indicating whether the sample was collected and the time the sample was collected.

Blood was drawn from the recruited study participants based on guidelines given by institutional review board (IRB) which requires blood to be drawn based on age and weight of a child. Venous blood amounting to 1ml was drawn from the eligible participants aged less than a year and 2 ml from the children aged one to five years. The blood was collected into serum separator tubes then labelled with the patient’s identification number and collection date. The blood was then stored in a cool box and transported to center for microbiology research (CMR) Kenya medical research institute (KEMRI) labs where it was centrifuged at 3000 rpm for three minutes to separate the serum. Using a pipette, the serum was transferred immediately into sterile cryovials then stored at -80°C until the time to run the ELISA.

### Laboratory procedure

#### Qualitative measurement of Ig G, Ig M and Ig A using ELISA

Detection of Autoantibodies against *Salmonella* Typhimurium and *Salmonella* Enteritidis in human serum was performed using IMTEC- *Salmonella*- Antibodies screen (Cut-off) (Szabo-Scandic HandelsgmbH, Vienna, Austria) ELISA kit. The protocol described in this peer-reviewed article has been submitted for publication on protocols.io and is included for printing as S1 Appendix in this article. The IMTEC- *Salmonella*- Antibodies screen (Cut-off) ELISA kit was a combined kit testing for IgA, IgG and IgM and also did not specify whether the antibodies were against *Salmonella* Typhimurium or *S*. Enteritidis. We opted to use IMTEC- *Salmonella*- Antibodies screen (Cut-off) ELISA kit since it is the only available kit for testing antibodies against NTS and it could detect any stage of exposure. The IMTEC company reports the kit to have a good sensitivity and specificity range of 91.7% and 94% respectively. The kit was also user friendly and did not require coating reagents which were unaffordable due to the limited funds. Hundred microliter of diluted serum sample, cut-off control (CC), positive control and negative control was dispensed into the *S*. Typhimurium and *S*. Enteritidis LPs precoated microtiter plate in the appropriate wells. The plate was then sealed and incubated at 37°C for 1 hour. After incubation, the solution was discarded and the plate washed three times using 300μl phosphate buffered saline (PBS) wash buffer. The wash buffer was discarded and residues knocked out on an absorbent paper. Hundred microliter of horse raddish peroxidase (HRP) conjugate solution was added. The plate was sealed then incubated for 30 minutes at room temperature. After incubation, the solution was discarded from the plate and then the plate was washed 3 times using PBS. The excess wash buffer was knocked out on an absorbent paper. Hundred microliter of substrate (tetramethyl benzidine) was pipetted on to all the wells then incubated for 10 minutes at room temperature. Immediately after the ten minutes, 100 μl of stop solution was added to all the wells then absorbance values were read within the next 10 minutes. The absorbance values were read at 450nm with a refence wavelength of 620-650nm using an ELISA reader BioTek ELX808. The results were interpreted as per the protocol where absorbances > 1.1 x CC were considered as positive antibody level and absorbances < 0.9 x CC were considered as negative.

### Data management and analysis

All parents or guardians of participating children were informed of the study objectives and voluntary written consent was sought and obtained before inclusion. A copy of the signed consent was filed and stored in locked cabinets at KEMRI. The data was analyzed using R- Software. Descriptive analysis was performed to aid describe the study population. Pearson’s Chi-squared test was performed in order to determine which population between the apparently healthy and patients is more exposed to NTS. A difference was considered statistically significant when the p- value was less than 0.05 (p<0.05). Multivariate logistic regression analysis was used to help determine the NTS associated host and environmental factors. Alpha level used was 0.05. Model was tested for multicollinearity and collinear variables did not seem to affect the predictive power of the model significantly. The variables were selected by backward stepwise elimination, where all the variables were initially included in the model and then variables were sequentially removed based on the p-value.

### Ethical consideration

This study was carried out on a protocol approved by the Scientific and Ethics Review Unit (SERU) of KEMRI (SERU No. 4331). Approval was also obtained from the administration of the hospitals where the study was conducted and from National Commission for Science, Technology and Innovation (NACOSTI).

## Results

### Demographic characteristics of the study population

Of the 382 study participants, those aged 25–36 months were found to have the highest number of participants in the study representing 22.25% while the age with least number of respondents was 49–60 months representing 17.28% of the entire sample (Table 1)

**Table 1:** Demographic characteristics of the study population.

Age	Male	Female	Total
0–12	38	35	73
	19.49	18.72	19.11
13–24	44	40	84
	22.56	21.39	21.99
25–36	43	42	85
	22.05	22.48	22.25
37–48	33	41	74
	16.92	21.93	19.37
49–60	37	29	66
	18.99	15.51	17.28
Total	195	187	382
Proportion (%)	51	49	100

### Seroprevalence of nontyphoidal *Salmonella* among apparently healthy children

Those participants found to be exposed to NTS were 24 while those who were not exposed were 167. The seroprevalence of NTS among the apparently healthy was 12.6% and the proportion of those who were not exposed to NTS were 87.4%. The proportion of the NTS exposed participants was significantly different from the proportion of those who were not NTS exposed (χ^2^ = 107.06; p = < 2.2×10^−16^). The study observed that, among the apparently healthy participants, 12.6% of the male were found to be exposed and 12.5% of the females were exposed. The differences in exposure to NTS among the female and male participants was not statistically significant (χ^2^ = 3.0473×10^−31^; p = 1). The mean age of those children who were exposed was 36 months and those not exposed was 27 months. Mean age of those children exposed to NTS and those not exposed was significantly different (W = 2646.5; p = 0.01121).

### Seroprevalence of nontyphoidal *Salmonella* among patients

Among the recruited patients, 24 participants were found to be exposed to NTS while those found not to be exposed were 167. The seroprevalence of NTS among the patients was 12.6% and the proportion of those who were not exposed to NTS were 87.4%. The proportion of the NTS exposed participants was significantly different from the proportion of those who were not NTS exposed (χ^2^ = 107.06; p = < 2.2×10^−16^). The study observed that, among the patients, 11.1% of the males were exposed and for the females, 14.5% were exposed. The differences in exposure to NTS among the male and female participants were not statistically significant (χ^2^ = 0.22233; p = 0.6373). The mean age of those children who were exposed was 39 months and those not exposed was 30 months. The difference in mean age of those children exposed and those not exposed to NTS was statistically significant (W = 2679; p = 0.0077).

#### Seroprevalence of nontyphoidal *Salmonella* among the apparently healthy and patients

Those participants found to be exposed to NTS were 48 while those who were not exposed were 334. The seroprevalence of NTS was 12.6% and the proportion of those who were not exposed to NTS were 87.4%. The proportion of the NTS exposed participants was significantly different from the proportion of those who were not NTS exposed (χ^2^ = 214.13; p < 0.05). The study observed that, the seroprevalence of NTS among male was 47.9% and for the female it was 52.0%. The seroprevalence of NTS among the female was not significantly different from the seroprevalence of NTS among the male participants (χ^2^ = 0.083333; p = 0.7728). The proportion of male who were not exposed to NTS was 51.5% and that of female was 48.50%. The observed difference among the female and male who were not exposed to NTS was not statistically significant (χ^2^ = 0.2994; p = 0.5843). The mean age of the study participants was 29 months. The average age of the exposed children was 38 months and that of those who were not exposed was 27 months. Mean age of those children exposed to NTS and those not exposed was significantly different (W = 9289.5; p< 0.05).

**Fig 1:**
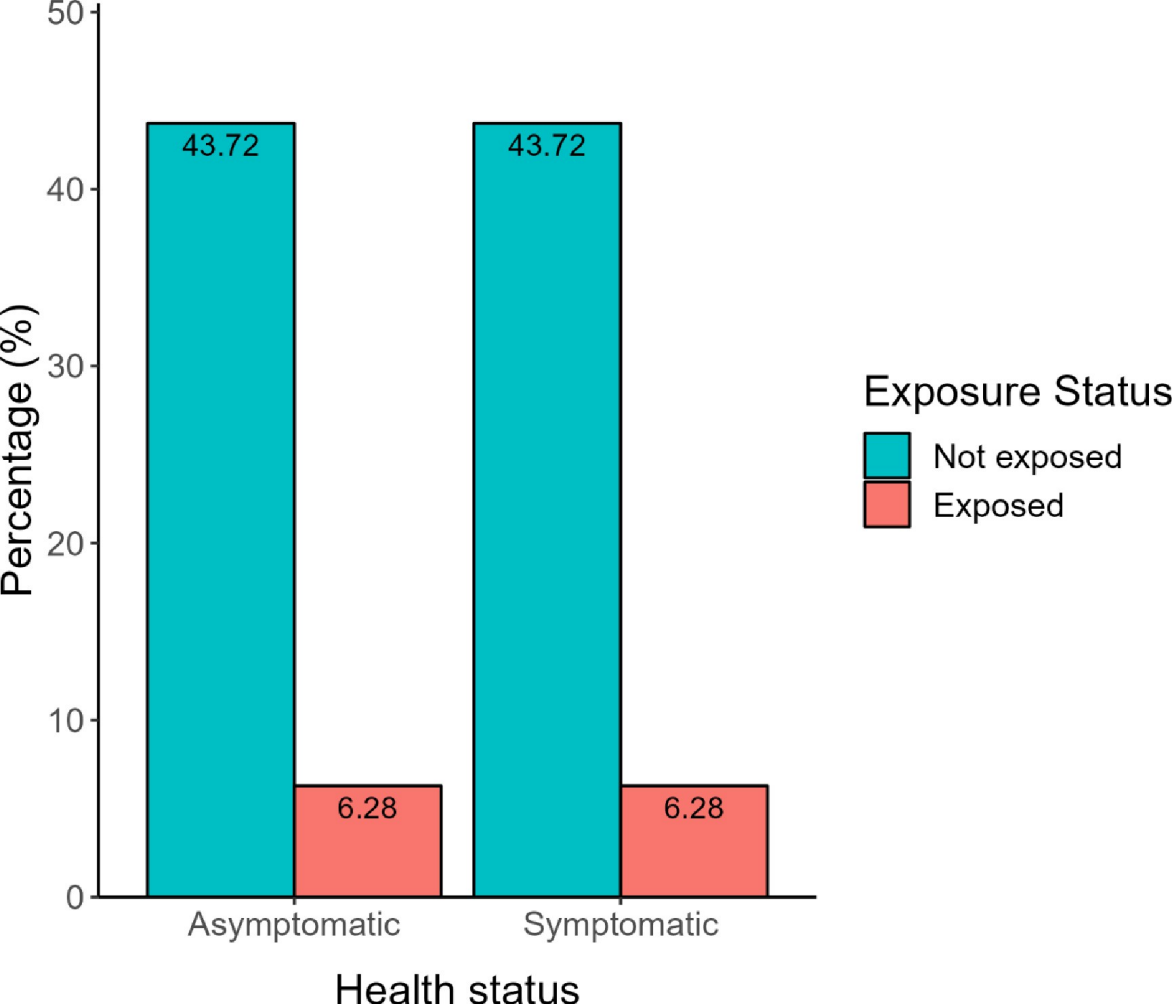
Exposure status of the apparently healthy and patients participants.

Out of the 191 apparently healthy participants, 24 were found to be exposed to NTS through ELISA and out of the 191 patients, 24 were also found to be exposed to NTS. The proportion of those who were exposed to NTS among the apparently healthy participants was not significantly different to the proportion of those who were exposed among the patients as analyzed using chi-square test (χ^2^ = 0.99649; p = 0.3182) (Fig 1).

#### Antibody titers of the study participants

The mean of the antibody titer for the apparently healthy population was 191.93 and that of the patients was 188.42. The observed difference in the mean of the antibody titer of the two populations was tested whether it was statistically significant using T-test and found not to be significant (p>0.05).

#### Spatial distribution of the NTS exposed participants

Out of the 24 apparently healthy exposed participants, 16 were from Mukuru kwa Njenga while 8 were from Mukuru kwa Ruben and among the patients, 24 of who were found to be exposed, 16 of them were from Mukuru kwa Njenga while 8 were from Mukuru kwa Ruben.

#### Host and environmental factors associated with NTS exposure

Keeping animals was significantly associated with NTS. Most common animals were chicken which accounted for 13.09%. Majority of the households were found to use the public municipal tap water 65.97% (Table 2). 51% of the households never treated water before drinking while 49% treated drinking water through boiling or use of chlorine. Majority of the participants (89.79%) used public toilets while 10.21% used private toilets.

**Table 2:** Distribution of homesteads by specific domestic animal kept, water, hygiene, sanitation.

Variable	N = 382	%
**Source of cooking water in the household**		
Own tap	3	0.79
Public municipal tap	252	65.97
Private tap	108	28.27
Public municipal borehole	16	4.19
Private borehole	3	0.79
Own private borehole	0	0
**Source of washing Water**		
Own tap	3	0.79
Public municipal tap	252	65.97
Private tap	108	28.27
Public municipal borehole	16	4.19
Private borehole	3	0.79
Own private borehole	0	0
**Water generally treated using**		
Boiling	79	20.68
Water guard	107	28.01
Other methods	0	0
**Keep animals**		
Chicken	50	13.09
Cattle	24	6.28
Goat	16	4.19
Pigs	13	3.40
Sheep	13	3.40

#### Factors associated with NTS exposure among the apparently healthy participants

All associated host and environmental factors (p- value <0.05) were analyzed using multivariate logistic regression (Table 3). Exposure to *Salmonella* Typhimurium and *Salmonella* Enteritidis was significantly associated with age of the child (p = 0.007), cooking water (p = 0.0.0368) and washing water (p = 0.0418). Treating water using chlorine or boiling method (p = 0.0449) was identified as being protective against contracting *Salmonella* Typhimurium and *S*. Enteritidis.

**Table 3:** Predisposing factors associated with NTS exposure in apparently healthy children as analyzed using multivariate logistic regression.

	Estimate	Std. Error	p-Value
(Intercept)	0.095005	0.064995	0.14551
Age in months	0.003951	0.001448	0.00697**
Cooking water	-0.211479	0.090494	0.03683*
Washing water	0.190285	0.0023917	0.04184*
Treated water	-0.095193	0.047149	0.04492*

Significant codes: 0 ‘***’ 0.001 ‘**’ 0.01 ‘*’ 0.05 ‘.’ 0.1 ‘ ‘ 1

#### Percentage contribution level by regressors to risk of exposure to iNTS disease

**Fig 2:**
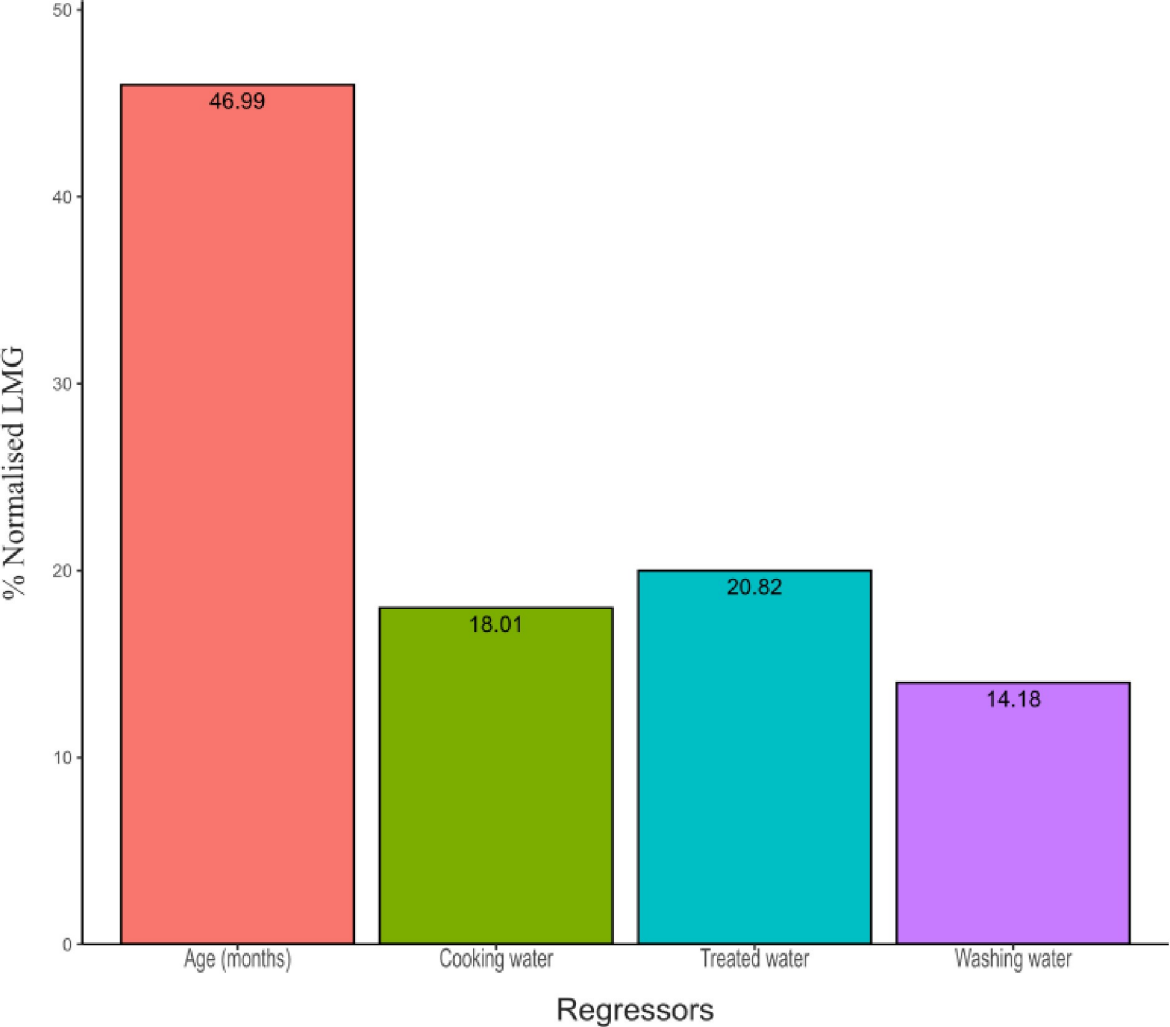
Percentage contribution of the regressors against results.

The percentage contribution of each of the regressors (independent variables) against the results was determined by use of normalized Lindeman, Merenda and Gold (LMG) measure. The LMG measure showed that, the highest contributor to the model was age 46.99% followed by treated water at 20.82% then cooking water at 18.01%. Washing water had the least contribution at 14.18%. The percentage contribution of the regressors is as illustrated in Fig 2.

#### Factors associated with NTS exposure among the patients

Exposure to iNTS among the patients was found to be significantly affected by keeping animals (p = 0.0239) and the chicken count (p = 0.00019). Keeping animals generally reduced the probability of disease occurrence while rearing chicken increased the disease occurrence Table 4.

**Table 4:** Predisposing factors associated with NTS exposure among sick children as analyzed using multivariate logistic regression.

	Estimate	Std. Error	p Value
(Intercept)	0.135593	0.029612	8.48e-06***
keep_animals	-0.123753	0.054339	0.023886*
chicken_count	0.019028	0.004995	0.000189***

Significant codes: 0

***’ 0.001

‘**’ 0.01

‘*’ 0.05 ‘.’ 0.1 ‘ ‘ 1

## Discussion

The proportion of children in Mukuru slums exposed to NTS was similar between the apparently healthy children and patients. The findings of the present study show a 12.6% seroprevalence of NTS among children aged 0–5 years in Mukuru informal settlement. To the best of our knowledge, this is the first Sero-epidemiology study that has ever been conducted in Kenya and the entire world and therefore no other done study to compare our findings.

Gender distribution of participants exposed to NTS showed a similar pattern between the male and female. This findings were different from a study conducted in Malawi and South Africa which found that females were more exposed to NTS than male [26] but similar to a study conducted in Uganda on cross-sectional study of IgG antibody levels to nontyphoidal *Salmonella* LPS O-antigen [27]. Age distribution of NTS indicated a peak at age 3.1–4.0 years where as lower proportion were observed among children aged 2.1–3.0 years. The seroprevalence of NTS among the apparently healthy and patients was found to be similar. This study results revealed that *Salmonella* Typhimurium and *S*. Enteritidis could occur in both symptomatic and asymptomatic state. This findings correspond to a study conducted in Nantong city which also revealed that many NTS could infect humans without any symptom [4]. Surveillance of *Salmonella* infections from both patients and apparently healthy people should be taken into consideration in order to control the disease.

The antibody titer of the apparently healthy participants was found to be significantly similar to that of the patients. The antibody titers among the healthy population implies that exposure to NTS infection is endemic in Mukuru informal settlement. This findings also implies that people in that region have developed immunity to NTS infections and also the children may have acquired the antibodies from their mothers through placental transfer.

The findings of this study showed a strong association between *Salmonella* Typhimurium and *S*. Enteritidis infections and host and environmental factors. A relevant point to note is that Mukuru informal settlement where this study was conducted is characterized by poor sanitation, dense population and unreliable water supply [23]. These are elements that create an environment favorable for rapid dissemination of enteric infections and other sanitation-related pathogens through contaminated food and water [28, 29]. Among the apparently healthy participants, NTS exposure was found to be higher among participants who used public municipal tap as water source for washing compared to those who used own tap in the house. Cooking water was found to reduce the probability of getting exposed to NTS since it has to undergo boiling process which makes it safe. In the informal settlements, many houses are made of corrugated iron and getting a private tap water supply in one’s house is very rare. Residents prefer getting water from public municipal tap since its cheaper than buying from individuals selling from their house. The NTS infection could be attributed to contaminated containers used in fetching the water and the fact that many residents fetch water from those water sources hence facilitating the spread.

Treatment of drinking water through boiling method or use of water guard was found to be associated with the NTS exposure. Treating drinking water made it safe and therefore made it protective against contracting the NTS infections. Age of the child was associated with NTS exposure. The present study finding is in accord with a study conducted in the same region which found that, age was an important risk factor associated with the diarrhoeal illnesses [23].

According to the present study, keeping animals significantly reduced the probability of exposure to NTS. The present study findings are in accord to a study conducted in the same site which found that, the occurrence of NTS disease was not significantly associated with rearing any domestic animal [23]. The present study also found that, rearing chicken would increase the chances of getting exposed to NTS. Due to the limited space where people live, most people sleep in the same house with the chicken therefore increasing the chances of contamination.

## Conclusion

The study concluded that, there is higher population that has been exposed to NTS including controls within Mukuru informal settlement and therefore the disease is endemic in that region. These is raising a lot of concern both at governmental level and non-governmental organization on the ways and strategies to mitigate on the transmission of *Salmonella* Typhimurium and *S*. Enteritidis. People from that region have developed immunity to the disease and therefore, measure of antibody levels cannot be used to predict disease. Majority of the recruited patients presented with gastroenteritis symptoms. Among the apparently healthy population, public municipal tap water used for cooking and washing, treating water and age of the study participants were the factors associated with NTS exposure. Among the patients, keeping animals and animal count were found to be associated with NTS exposure. According to these study, there is need for increased surveillance of NTS from both patients and apparently healthy individuals in order to help control the disease. Sensitization of people is required in order to help them understand the transmission modes and inform of the ways that can help reduce their exposure to NTS.

## Supporting information

S1 File Clinical examination data form. (DOCX)

S2 File Apparently healthy questionnaire data. (XLSX)

S3 File Patients questionnaire data. (XLSX)

S1 Table Apparently healthy ELISA data. (XLSX)

S2 Table Patients ELISA data. (XLSX)

S1 Appendix Step-by-step protocol, also available on protocols.io. (DOCX)
