# Supplementary material for: Seroprevalence of non-typhoidal Salmonella disease and associated factors in children in Mukuru settlement in Nairobi County, Kenya
Source: PLoS One. 2023 Jul 17;18(7):e0288015. doi: 10.1371/journal.pone.0288015 (PMC10351689; doi:10.1371/journal.pone.0288015)
Supplement: S1 File — (DOCX) [file pone.0288015.s001.docx]

**Questionnaire to investigate factors for invasive non-typhoidal *Salmonella* disease in children living in Mukuru informal settlement**

**Assessment of exposure to *Salmonella* Enteritidis and *S.* Typhimurium O antigen among a population aged 0-5 years attending Mukuru clinics in Nairobi County.**

**Please answer ALL questions appropriately**

1. Name of the field worker/Nurse_______________________

2. Date: // (DD/MM/YYYY)

3. Has parent/guardian provided informed consent for child’s participation?

○ Yes ○ No

A. If YES, provide date of Informed consent:

// (DD/MM/YYYY)

**GENERAL INFORMATION**

3. Who is the respondent?

○ Mother ○ Father ○ Guardian ○ Other

4. Name of child/participant: _____________________________________________

5. Phone number where participant /parent/legal guardian can be contacted:

A. First phone number:

B. Second phone number (if necessary):

6. Is the Date of Birth of your child fully or partially known?

○ Complete (Day-Month-Year) ○ Partially complete (Year Only)

○ Partially complete (Month-Year) ○ Unknown

A. Date of birth of child (if known):

If COMPLETE, provide date: // (DD/MM/YYYY)

If PARTIALLY COMPLETE, specify month and year: / (MM/YYYY)

If PARTIALLY COMPLETE, specify year: (YYYY)

Age of child __ __ months/years (if DOB is unknown)

|  | **STUDY INCLUSION CRITERIA** | Yes | No |
| --- | --- | --- | --- |
|  | Is the participant ≤ 5 years of age? | ○ | ○ |
|  | Does the participant reside in this area? | ○ | ○ |
|  | Does the parent/guardian agree to have blood samples collected from their child?  ○ Yes ○ No |  | |
|  | If NO to any of the above, DO NOT proceed with CRF | | |

**STUDY EXCLUSION CRITERIA**

1. Is the child asymptomatic for invasive non-typhoidal salmonella disease as judged by the study team?

○ Yes ○ No

1a) If no in question one above, did you have fever in the past 3 days?

○ - Yes ○ 0 - No ○ 99 – Unknown

1b) Did you take any medication in the last 8 weeks?

0Yes 0No 0not sure

If yes, which medication did you take?

| Chloramphenicol Amoxicillin Co-Amoxiclav Ciprofloxacin Ceftriaxone  Anti-malarial drugs Others, specify_______________ Not applicable  Don’t know |
| --- |

1c) Does the subject have any past or current symptom of the following in past 4 weeks?

|  |  | Yes | No | DK |  | Yes | No | DK |
| --- | --- | --- | --- | --- | --- | --- | --- | --- |
| Respiratory | Cough |  |  |  | Expectoration |  |  |  |
|  | Hemoptysis |  |  |  |  |  |  |  |
| G-I | Vomiting |  |  |  | Diarrhea |  |  |  |
|  | Abdominal Pain |  |  |  | Blood diarrhea |  |  |  |
|  | Distension |  |  |  | Watery diarrhea/colour |  |  |  |
| Neurology | Headache |  |  |  | Seizures |  |  |  |

**SOCIO-ECONOMIC CHARACTERISTICS OF THE HOUSEHOLD**

11. Please think about the person who is the head of your household. It maybe you, or it may be someone else. What is the highest educational qualification of the household head? Choose:

○ Never been to school ○ College/ University/Tertiary

○ Primary ○ Unwilling to answer

○ Secondary ○ Uknown

12. Is household head able read and write?

○ Yes ○ No ○ Unwilling to answer ○ Unknown

13. How many people live in your household? Please count all the people who normally live with you and eat meals together. Include yourself when counting.

13a. How many children less than 5 years of age are there in the household?

14. In the past 7 days, did you worry that your household would not have enough food?

○ Yes ○ No ○ Unknown

15. Concerning your housing (structure, form and type), which of the following is true?

○ It is less than adequate for Household needs ○ It is more than adequate for Household needs

○ It is just adequate for Household needs ○ Unknown

16. Concerning your household’s clothing, which of the following is true?

○ It is less than adequate for Household needs ○ It is more than adequate for Household needs

○ It is just adequate for Household needs ○ Unknown

1. Imagine six steps, where on the bottom, the first step, stand the poorest people, and on the highest step, the sixth, stand the rich. Show the picture of the steps. On which step are you today?

○ Step 1 ○ Step 2 ○ Step 3 ○ Step 4

○ Step 5 ○ Step 6 ○ Unknown

1. Does your household own a bed?

○ Yes ○ No ○ Unknown

1. What does the head of the household sleep on? Training (what is the best bed in the house?)

○ Bed frame and Mattress ○ Cloth/Sack on floor

○ Bed frame and Mat (grass) ○ Floor (nothing else)

○ Bed frame alone ○ Other (Specify: ­­­­­­­­­­­­­­­­­____________________________

○ Mattress on floor ○ Unknown

○ Mat (grass) on floor

1. Does your household own a table?

○ Yes ○ No ○ Unknown

1. Do you, either by yourself or together with another household member or someone outside your household, currently have an account at a bank, credit union, micro finance institution, village savings organization, or another financial institution?

○ Yes ○ No ○ Unknown

1. Do you have working electricity in your dwelling?

○ Yes ○ No ○ Unknown

1. Does your household own an upholstered chair (arm chair) or sofa set?

○ Yes ○ No ○ Unknown

**ANIMAL OWNERSHIP**

1. Does your household keep any animals? ○ Yes ○ No

If YES, is it…

1. Companion pet/animal (e.g. Dog, cat etc…): ○ Yes ○ No
2. Livestock (e.g. cattle, chicken, goat etc…): ○ Yes ○ No
3. If YES to Q22(B), of the following, how many do you have?

○ Chicken: Number: ____________________

○ Cattle: Number: ____________________

○ Goat: Number: ____________________

○ Pigs: Number: ____________________

○ Sheep: Number: ____________________

○ Others (Specify): _______________________________________________

**WASH AND SANITATION**

25. Which source do you use most frequently to get water for cooking?

○ Public municipal tap ○ Your own private borehole (pump handle)

○ Private tap (individual selling water from their house) ○ Bottled water

○ Your own tap in your own house ○ Protected well

○ Public municipal borehole (pump handle) ○ Unprotected well

○ Private borehole (owned by an individual selling water) ○ River or stream or spring

(unprotected open water source)

27. Which source do you use most frequently to get water for washing clothes?

○ Public municipal tap ○ Your own private borehole (pump handle)

○ Private tap (individual selling water from their house) ○ Bottled water

○ Your own tap in your own house ○ Protected well

○ Public municipal borehole (pump handle) ○ Unprotected well

○ Private borehole (owned by an individual selling water) ○ River or stream or spring

(unprotected open water source)

○ Unknown

28. Which source do you use most frequently to get water for bathing?

○ Public municipal tap ○ Your own private borehole (pump handle)

○ Private tap (individual selling water from their house) ○ Bottled water

○ Your own tap in your own house ○ Protected well

○ Public municipal borehole (pump handle) ○ Unprotected well

○ Private borehole (owned by an individual selling water) ○ River or stream or spring

(unprotected open water source)

○ Unknown

29. Which source do you use most frequently for drinking?

○ Public municipal tap ○ Your own private borehole (pump handle)

○ Private tap (individual selling water from their house) ○ Bottled water

○ Your own tap in your own house ○ Protected well

○ Public municipal borehole (pump handle) ○ Unprotected well

○ Private borehole (owned by an individual selling water) ○ River or stream or spring

(unprotected open water source)

○ Unknown

30. Do you treat your drinking water before drinking?

○ Yes ○ No ○ Unknown

31. If yes to Q30, please specify how the drinking water is treated:

○ Use of water guard / bleach/ chlorine tablets ○ Solar disinfection

○ Boiling ○ Purchased water filter

○ Sieve through cloth ○ Others (Specify): _______________

○ Unknown

32. What type of toilet is used by your household?

○ Flush/pour flush ○ Open defaecation

○ Pit latrine with slab ○ Unwilling to answer

○ Pit latrine with wood/soil floor ○ Unknown

33. Who do you share the toilet facility with?

○ Household use only ○ Shared with more than 3 households

○ Shared with 3 households ○ Do not have one (open defecation) ○ Unknown

**PAST MEDICAL HISTORY**

34. Medical history taken from (allow multiple select):

○ Health card

○ Parental Recall

○ Unknown

| Vaccine history | Yes | No | Unknown |
| --- | --- | --- | --- |
| BCG | ○ | ○ | ○ |
| OPV 0 | ○ | ○ | ○ |
| OPV 1, DPT-HepB-Hib1, PCV1 & Rotavirus vaccine 1 | ○ | ○ | ○ |
| OPV 2, DPT-HepB-Hib2, PCV2 & Rotavirus vaccine 2 | ○ | ○ | ○ |
| OPV 3, DPT-HepB-Hib3 & PCV 3 | ○ | ○ | ○ |
| Measles and Rubella 1 | ○ | ○ | ○ |
| Measles and Rubella 2 | ○ | ○ | ○ |
| Additional vaccines?  Please Indicate: | ○ | ○ |  |

| Has your child had fever in the last month? | ○ Yes | ○ No | | ○ Unknown |
| --- | --- | --- | --- | --- |
| Has your child been tested for malaria in the last month? | ○ Yes | ○ No | | ○ Unknown |
| If yes, what was the result of your child’s malaria test? | ○ Positive | ○ Negative | | ○ Unknown |
| If the child tested positive for malaria, did they receive treatment? | ○ Yes | ○ No | | ○ Unknown |
| Is the child HIV exposed?(documentation of the HIV exposure in the H/card) | ○ Yes | ○ No | | |
| Has this child had an HIV test? | ○ Yes | ○ No | | ○ Unknown |
| When the child was last tested for HIV? (if yes to Q above) |  | |  | |
| What is the child's HIV status? | ○ Negative | | ○ Positive | |
| If positive, is the child on ART treatment? | Yes ○ No ○ | | | |
| Does your child have sickle cell disease? | ○ Yes | ○ No | | ○ Unknown |
| Has your child had anaemia in the last 12 months? | ○ Yes | ○ No | | ○ Unknown |
| Has your child been diagnosed with malnutrition in the last 12 months? | ○ Yes | ○ No | | ○ Unknown |

**GENERAL EXAMINATION**

|  | Axillary Temperature (all age categories) | _______ ^o^C |
| --- | --- | --- |
|  | MUAC | _______ cm |

**INVESTIGATIONS & RESEARCH SAMPLES**

|  | SAMPLES COLLECTED FOR RESEARCH | | | | |
| --- | --- | --- | --- | --- | --- |
|  | Sample | Sample Collection | Sample Volume: | Sample Collection Date *(DD/MM/YYYY)*  And Time (HH:MM) | Lab identifier |
| 53. | Blood | Yes ⬜  Yes but Inadequate ⬜  No ⬜ | ⬜⬜ mls | ⬜⬜/⬜⬜/⬜⬜⬜⬜  ⬜⬜ : ⬜⬜ |  |
|  | Stool | Yes ⬜  No ⬜ |  |  |  |

1. For blood sample, if Yes but inadequate /No to question 53, what is the reason for no sample collection:

○ Child has difficult veins

○ Unco-operative child

○ Sample spilt

○ Other (Specify): _________________________________________________
