# Supplementary material for: Seroprevalence of non-typhoidal Salmonella disease and associated factors in children in Mukuru settlement in Nairobi County, Kenya
Source: PLoS One. 2023 Jul 17;18(7):e0288015. doi: 10.1371/journal.pone.0288015 (PMC10351689; doi:10.1371/journal.pone.0288015)
Supplement: S1 Appendix — (DOCX) [file pone.0288015.s006.docx]

**Lab protocol**

**Seroprevalence of non-typhoidal *Salmonella* disease and associated factors in children in Mukuru settlement in Nairobi County, Kenya.**

**Metadata**

**Schola K. Peter ^1,2^ , Joshua M. Mutiso^1^, Mercy Ngetich^2^, Cecilia Mbae^2^, Samuel Kariuki^2,3^**

**Author affiliation**

1. Department of Zoological Sciences, Kenyatta University, Nairobi, Kenya
2. Centre for Microbiology Research, Kenya Medical Research Institute
3. Wellcome Trust Sanger Institute, Cambridge, UK

**Corresponding author Email** : [kamwethya14@gmail.com](mailto:kamwethya14@gmail.com) (SK)

**Funding**

The authors received no specific funding for this work.

**Competing interests**

The authors declare that there is no conflict of interest.

**Data availability**

Our study does not have any data that has links or code produced by the protocol or necessary to interpret the outputs.

**Associated content**

Not available

**Abstract**

IMTEC *Salmonella* antibodies screen (cut-off) is an ELISA used for invitro qualitative measurement of immunoglobulin (Ig) A, Ig G and Ig M autoantibodies against *Salmonella* Enteritidis *and S.* Typhimurium in human serum samples. The protocol is designed to detect Ig M typically appearing at the beginning of the Salmonella infection, Ig A which indicate persistence of the *Salmonella* infection in the intestines and Ig G which indicate a previous infection that is no longer active. The IMTEC antibodies screen (cut-off) kit neither distinguish between recent or past infections nor does it distinguish between the seropositivity of *S.* Typhimurium or *S.* Enteritidis. A sample was considered positive for Ig A, Ig G or Ig M if the absorbance value was greater than 1.1× cut-off control or negative if absorbance value was less than 0.9 × cut-off control. The absorbance values are determined using an ELISA reader. R software was used to determine seroprevalence and antibody titers of the samples.

**Introduction**

IMTEC *Salmonella* antibodies screen (cut-off) is an enzyme immunoassay (ELISA) developed for the invitro qualitative measurement of IgG, IgM and IgA autoantibodies against *Salmonella* Enteritidis or *S.* Typhimurium lipopolysaccharide (LPs) antigen in human serum. The ELISA kit uses high Ig A titers as a marker for the diagnosis of Salmonella- associated reactive arthritis. IMTEC *Salmonella* antibodies screen (cut-off) and results reported by Isoma¨ki et al. (1989), Seuri et al. (2005), Dalby et al. (2005) and Strid et al. (2007) uses mixture of *Salmonella* Enteritidis and *S.* Typhimurium (LPs). Some other reported assays used LPS from a single *Salmonella* serovar either *S.* Enteritidis or *S.* Typhimurium separately [1].

Another study used both mixed LPs and single LPs and concluded that, the two assays are equally very useful for routine analyses of human sera when gastrointestinal *Salmonella* infections are suspected and the bacteria are no longer shed [2]. An LPS-based ELISA which detects IgA, IgG and IgM responses is said to provide a highly specific, sensitive, rapid, simple and dependable assay for routine analyses of human sera [2]. Six studies reported sensitivity range between 78% - 100% and specificity range between 90% - 97% of the ELISA used while IMTEC anti-*Salmonella* ELISA has a sensitivity of 91.7% and a specificity of 94%. IMTEC anti-*Salmonella* ELISA is the only commercially available kit for diagnosis of non-typhoid human salmonellosis [1]. The IMTEC antibodies screen (cut-off) kit neither distinguish between recent or past infections nor does it distinguish between the seropositivity of *S.* Typhimurium or *S.* Enteritidis.

**Materials and methods**

Detection of Autoantibodies against *Salmonella* Typhimurium and *Salmonella* Enteritidis in human serum was performed using IMTEC- Salmonella- Antibodies screen (Cut-off) (Szabo-Scandic HandelsgmbH, Vienna, Austria) ELISA kit. Hundred microliter of diluted serum sample, cut-off control, positive control and negative control was dispensed into the microtiter plate in the appropriate wells. The plate was then sealed and incubated at 37°C for 1 hour. After incubation, the solution was discarded and the plate washed three times using 300µl phosphate buffered saline (PBS) wash buffer. The wash buffer was discarded and residues knocked out on an absorbent paper. Hundred microliter of horse raddish peroxidase (HRP) conjugate solution was added. The plate was sealed then incubated for 30 minutes at room temperature. After incubation, the solution was discarded from the plate and then the plate was washed 3 times using PBS. The excess wash buffer was knocked out on an absorbent paper. Hundred microliter of substrate ( tetramethyl benzidine) was pipetted on to all the wells then incubated for 10 minutes at room temperature. Immediately after the ten minutes, 100 µl of stop solution was added to all the wells then absorbance values were read within the next 10 minutes. The absorbance values were read at 450nm with a refence wavelength of 620-650nm using an ELISA reader BioTek ELX808.

**Expected results**

The expected results were either negative or positive IgA, IgG and IgM which was determined by the absorbance values obtained. Interpretation of results was made by comparing the absorbances of cut-off control (CC) and serum samples. That is Absorbances > 1.1 x (CC) were considered as positive and absorbances < 0.9 x (CC) were considered as negative. To the best of our knowledge, this is the first Sero-epidemiology study that has ever been conducted in Kenya and the entire world using this ELISA kit and therefore no other done study to compare our data with.

**Ethics declaration**

This study was carried out on a protocol approved by the Scientific and Ethics Review Unit (SERU) of KEMRI (SERU No. 4331). Approval was also obtained from the administration of the hospitals where the study was conducted and from National Commission for Science, Technology and Innovation (NACOSTI).

**Supporting information**

S1: Step-by-step protocol, also available on protocols.io

**Acknowledgements**

The authors sincerely acknowledge the field and laboratory staff of the Mukuru kwa Njenga and Mukuru kwa Ruben invasive nontyphoidal *Salmonella* disease surveillance project for their contribution in this study.

**Authors contribution**

**Schola K. Peter:**  Conceptualization, Data curation, Formal analysis, Methodology, Project administration, Writing original draft, editing review comments

**Joshua M. Mutiso:**  Data curation, Investigation, Methodology, Project administration, Supervision

**Samuel Kariuki:** Project administration, funding acquisition, review and editing

**References**

1. Kuhn KG, Falkenhorst G, Ceper TH, Dalby T, Ethelberg S, Mølbak K, et al. Detecting non-typhoid Salmonella in humans by ELISAs: a literature review. J Med Microbiol. 2012 Jan 1;61(1):1–7. DOI 10.1099/jmm.0.034447-0

2. Strid MA, Dalby T, Mølbak K, Krogfelt KA. Kinetics of the Human Antibody Response against *Salmonella enterica* Serovars Enteritidis and Typhimurium Determined by Lipopolysaccharide Enzyme-Linked Immunosorbent Assay. Clin Vaccine Immunol. 2007 Jun;14(6):741–7. DOI:10.1128/CVI.00192-06
